# Supplementary material for: The mesh is a network of microtubule connectors that stabilizes individual kinetochore fibers of the mitotic spindle
Source: eLife. 2015 Jun 19;4:e07635. doi: 10.7554/eLife.07635 (PMC4495718; doi:10.7554/eLife.07635)
Supplement: Source code 1. — Custom written code in IgorPro 6.36 (Wavemetrics) was used for all analysis and plotting. DOI: http://dx.doi.org/10.7554/eLife.07635.020 [file elife07635s001.rtf]

Code for MT analysis in Nixon et al., 2015.The following procedures were used with MT co-ordinate sets using Igor Pro 6.3x (Wavemetrics). The following code can be used as a *.ipf to perform similar analyses.#pragma rtGlobals=3		// Use modern global access method and strict wave access.#include <All Gizmo Procedures>//This function will calculate the cross-sectional area of a MT bundle//It will also count the MTs in the bundle and work out the density of MTs/area//Requires 2 x 1D waves one with x co-ords, 1 with y co-ords Function AreaCalc(theXList,theYlist)	String theXList	String theYList		Variable i=0	string aWaveName = ""	string bWaveName = ""	Variable nsize=ItemsInList(theXlist)	Make /O /T /N=(nsize) TextWave0	//this will be a list of wavenames	Make /O /N=(nsize) areaWave		//this will contain the cross sectional areas	Make /O /N=(nsize) MTWave			//this will contain the number of MTs	Wave W_Xhull,W_Yhull	do		aWaveName = StringFromList(i, theXList)		bWavename = StringFromList(i, theYList)		WAVE/Z aWave = $aWaveName		WAVE/Z bWave = $bWaveName		if (!WaveExists(aWave))			break		endif			TextWave0[i]=aWaveName			ConvexHull /C aWave,bWave			areaWave[i]=polygonarea(W_Xhull,W_Yhull)			MTWave[i]=numpnts(aWave)		i += 1	while(1)	duplicate /o MTWave DensityWave	Densitywave /=areaWave	//calculate density	edit Textwave0,MTWave,AreaWave,DensityWaveEnd//This function will calculate the distance to the nearest neighbour for each MT//Requires 2 x 1D waves one with x coordinates, 1 with y coordinates Function NNFinder(theXList,theYlist)	String theXList	String theYList		String xWave, yWave	Variable nWaves	Variable i	//waveindex	Variable j	//1stMT	Variable k	//rowindex	Variable ncr	//combinations to be evaluated		nWaves = ItemsInList(theXList)	ncr=nwaves*(nwaves-1)	Make /O /N=(ncr) TempWave	Make /O /N=(nWaves) DistWave		for (i = 0; i < nWaves; i += 1)	xWave = StringFromList(i, theXlist)	yWave = StringFromList(i, theYlist)	Wave/Z xw = $xWave	Wave/Z yw = $yWave		for (j = 0; j < numpnts(xw); j+=1)		k=0		if (k==j)			TempWave[k]=nan		else			TempWave[k]=sqrt(((xw[j]-xw[k])^2)+((yw[j]-yw[k])^2))			k+=1		endif			WaveStats /Q TempWave			DistWave[i]=V_min		endfor	endforEnd//This function will take a list of x and y waves and go through them for a variety of sizes //send to HowMany() to work out the number of neighbours within a range (of a given interval)Function Send(theXList,theYlist,nmMin,nmMax,step)	String theXlist	//wavelist of waves containing x co-ords in nm	String theYlist	//wavelist of waves containing y co-ords in nm	Variable nmMin	//minimum search radius e.g. 20	Variable nmMax	//maximum search radius e.g. 120	Variable step	//interval size for search radii e.g. 10 (20, 30,...,110,120)		Make /o /N=(((nmmax-nmmin)/step)+1) StepWave	StepWave =(x*step)+nmmin		Variable i,s		//loop variables	String xWave	String yWave		for (i = 0; i < ItemsInList(theXlist); i += 1)	xWave = StringFromList(i, theXlist)	yWave = StringFromList(i, theYlist)		for  (s = 0; s < numpnts(StepWave); s +=1)		HowMany($xWave,$yWave,StepWave[s])		endfor	endfor	KillWaves StepWaveEnd//This function finds how many neighbouring MTs are within x nm of each MTFunction HowMany(xW,yW,nm)	Wave xW	Wave yW	Variable nm		//search radius		Variable mts		//number of MTs in the co-ordinate set	Variable j 		//1stMT	Variable k 		//2nd MT	Variable l 		//rowindex of TempWave//	Variable ncr 	//combinations to be evaluated		mts = numpnts(xW)//	ncr=mts*(mts-1)	Make /O /N=(mts) TempWave	Make /O /N=(mts) NeighbourWave	NeighbourWave =0	String newname = ReplaceString("x_",NameofWave(xW),"nm_")+"_"+num2str(nm)		for (j = 0; j < mts; j+=1)		//for each MT in fiber find all cartesian distances		for (k = 0; k < mts; k+=1)			TempWave[k]=sqrt(((xw[j]-xw[k])^2)+((yw[j]-yw[k])^2))	//store distances here		endfor				for (l = 0; l < mts; l += 1)	//go through distances, count those <= search radius			if (TempWave[l]== 0)		//does not count 0 nm distances, i.e MT 			elseif (TempWave[l]<=nm)			NeighbourWave[j] +=1			endif		endfor	endfor	Rename NeighbourWave $newnameEnd//Procedure to randomize bch waveFunction Randomize(waveNames)	String waveNames		//wavelist of bch waves for randomisation		Variable i	For (i = 0; i < ItemsInList(waveNames);i +=1)		String name = StringFromList(i,waveNames)		Wave /z w1 = $name		Make/n=(numpnts(w1))/o keyw=abs(enoise(1))		Sort keyw w1	EndFor	Killwaves keywEnd//Function to concatenate all values into one long wave.//For each search radius for each fibre, a 2D wave was made with branch membership data as 1st column and number of neighbours as 2nd//These 2D waves were concatenated by this functionFunction Concat(root,w1Name)	// call example: Concat("nn_Ctrl*","Ctrl_all")	String root				//prefix of waves	String w1Name			//target wave		String list=wavelist(root,";","")	Concatenate /o /NP=0 list, $w1Name	//creates new wave or overwrites existingEnd//This is a simple gating function. Run on 2D waves and specify a "level", i.e. cut off for gating//The function will generate two waves lo and hi for w1 and then make a frequency histogram of both Function Splitter(w1,level) 	wave w1	variable level				// level=1 for differentiating singles from branches		Variable size =dimsize(w1,0)	//size of one column	string waveloname=NameofWave(w1)+"lo"	string wavehiname=NameofWave(w1)+"hi"	duplicate /o w1 $waveloname	//these waves will be the new split waves	duplicate /o w1 $wavehiname	wave wavelo=$waveloname	wave wavehi=$wavehiname		Variable rowIndex			//the loop variable   	for (rowIndex = 0; rowIndex < size; rowIndex += 1)		if (w1[rowIndex][0]>level)		wavelo[rowindex][]=nan		endif		if (w1[rowIndex][0]<=level)		wavehi[rowIndex][]=nan		endif	endfor	string newloname=NameofWave(w1) + "lo2"	string newhiname=NameofWave(w1) + "hi2"	Duplicate/O/R=[][1] wavelo, tempwave		//makes a copy of the 1st wave (but this is still multi-dimensional, so...)	Extract tempwave, $newloname, tempwave>=0	// equivalent of zapnans into a new wave	Duplicate/O/R=[][1] wavehi, tempwave		//makes a copy of the 1st wave (but this is still multi-dimensional, so...)   	Extract tempwave, $newhiname, tempwave>=0	// equivalent of zapnans into a new wave	killwaves tempwave						//clean up	string newlohistname=NameofWave($newloname) + "_Hist"	string newhihistname=NameofWave($newhiname) + "_Hist"	Make/N=12/O $newlohistname				//use this to make a histogram	Histogram/P/B={0,1,12} $newloname, $newlohistname	Make/N=12/O $newhihistname				//use this to make a histogram	Histogram/P/B={0,1,12} $newhiname, $newhihistname	display $newhihistname	appendtograph $newlohistname	ModifyGraph mode=5	ModifyGraph hbFill($newlohistname)=4End//This function makes MT waves - these are 2D waves (3 columns = x y z, 2 rows = start end)//The input is from excel where each column is a MT rows are: MT number, startx,starty,startz,endx,endy,endz//MTmaker will make MT waves from this list.Function MTmaker(wavenames)	String wavenames	//this will be wavelist("wave*",";","")	String name  	Variable i  //loop Variable	for (i = 0; i < ItemsInList(wavenames); i += 1)	name = StringFromList(i,wavenames)	Wave w1=$name	string MTwave="MT" + num2str(w1(0))	Make /O $MTwave={{w1(1),w1(4)},{w1(2),w1(5)},{w1(3),w1(6)}}	endforEnd//This function compares the angles of MTs relative to one another.//It doesn't compare a given MT with itself nor are there replications - handshake problem//The result is always positive, so it tells you about deviation from the MT.//Result is in degrees (*180/pi). Only results from 0-180 are possible//In reality not much beyond 90 is possible because it finds the most acute angle//MTs all go in the same direction so this constrains things further//This method was not used in the paperFunction MTpairer(WavesList)	String WavesList	String Wave1, Wave2, pair	Variable nWaves, nsize	Variable i, j, k	Variable ABx, CDx, ABy, CDy, ABz, CDz		nWaves = ItemsInList(WavesList)	nsize = (nWaves*(nwaves-1))/2	Make /O /T /N=(nsize) LabelWave	Make /O /N=(nsize) AngleWave		for (i = 0; i < nWaves; i += 1)	Wave1 = StringFromList(i, WavesList)	Wave/Z w1 = $Wave1		for (j = 0; j < nWaves; j+=1)		Wave2 = StringFromList(j, WavesList)		Wave/Z w2 = $Wave2			if (j>i)			pair=Wave1 + "_" + Wave2			LabelWave[k]=pair			ABx=w1[1][0]-w1[0][0]			CDx=w2[1][0]-w2[0][0]			ABy=w1[1][1]-w1[0][1]			CDy=w2[1][1]-w2[0][1]			ABz=w1[1][2]-w1[0][2]			CDz=w2[1][2]-w2[0][2]			AngleWave[k]=acos(((ABx*CDx)+(ABy*CDy)+(ABz*CDz))/(sqrt((ABx^2)+(ABy^2)+(ABz^2))*sqrt((CDx^2)+(CDy^2)+(CDz^2))))*(180/pi)			k+=1			endif		endfor	endforEnd//MTcomparer compares every MT with a reference MT called refMT//RefMT is created from finding the centre of the bundle at the start and at the end//Otherwise this function is the same as MTpairer.//It actually compares refMt to each MT and not the other way around - but this doesn't matter//This method was not used in the paperFunction MTcomparer(WavesList)	String WavesList	String Wave1, pair	Wave/Z w2 = RefMT	Variable nWaves	Variable i	Variable ABx, CDx, ABy, CDy, ABz, CDz		nWaves = ItemsInList(WavesList)	Make /O /T /N=(nWaves) RefLabelWave	Make /O /N=(nWaves) RefAngleWave	Make /O /N=(nWaves) RefDistWave		for (i = 0; i < nWaves; i += 1)	Wave1 = StringFromList(i, WavesList)	Wave/Z w1 = $Wave1			pair="RefMT_" + Wave1 			RefLabelWave[i]=pair			ABx=w1[1][0]-w1[0][0]			CDx=w2[1][0]-w2[0][0]			ABy=w1[1][1]-w1[0][1]			CDy=w2[1][1]-w2[0][1]			ABz=w1[1][2]-w1[0][2]			CDz=w2[1][2]-w2[0][2]			RefAngleWave[i]=acos(((ABx*CDx)+(ABy*CDy)+(ABz*CDz))/(sqrt((ABx^2)+(ABy^2)+(ABz^2))*sqrt((CDx^2)+(CDy^2)+(CDz^2))))*(180/pi)			//calculate distances			RefDistWave[i]=sqrt(((w1[0][0]-w2[0][0])^2)+((w1[0][1]-w2[0][1])^2))	endforEnd//find spherical coordinates for each MTFunction SpherCoord([rot]) 	//use () to calculate original MTs, use (rot=1) for rotated MTs	Variable rot		String wList	If (paramisdefault(rot)==1	)		wList=wavelist("MT*",";","")	else		wList=wavelist("rMT*",";","")	endif			String wName,wSC	Variable nWaves	Variable i	Variable wx, wy, wz		string LabelWName, rWName, thetaWName, phiWName		If (paramisdefault(rot)==1	)		LabelWName="SClabelWave"		rWName="SCrWave"		thetaWName="SCthetaWave"		phiWName="SCphiWave"	else		LabelWName="rSClabelWave"		rWName="rSCrWave"		thetaWName="rSCthetaWave"		phiWName="rSCphiWave"	endif		nWaves = ItemsInList(wList)	Make /O /T /N=(nWaves) $LabelWName	Make /O /N=(nWaves) $rWName	Make /O /N=(nWaves) $thetaWName	Make /O /N=(nWaves) $phiWName	Wave /T LabelW=$LabelWName	Wave rW=$rWName	Wave thetaW=$thetaWName	Wave phiW=$phiWName	for (i = 0; i < nWaves; i += 1)	wName = StringFromList(i, wList)	Wave/Z w = $wName			wSC =wName 			LabelW[i]=wSc						wx=w[1][0]-w[0][0]			If  ((wx > -0.001) && (wx < 0.001))	//if statements to prevent errors from rounding				wx=0			endif			wy=w[1][1]-w[0][1]			If  ((wy > -0.001) && (wy < 0.001))				wy=0			endif						wz=w[1][2]-w[0][2]			If  ((wz > -0.001) && (wz < 0.001))				wz=0			endif			rW[i]=sqrt((wx^2)+(wy^2)+(wz^2))			thetaW[i]=acos(wz/(sqrt((wx^2)+(wy^2)+(wz^2))))			//need to *(180/pi) to get °						phiW[i]=atan2(wy,wx)	endfor	Variable V_avg,V_sdev	Wavestats /q rW	Print "Radial distance, r: mean",V_avg,"±",V_sdev,"nm. Median =",Statsmedian(rW)	Wavestats /q thetaW	Print "Polar angle, theta: mean",V_avg,"±",V_sdev,"radians. Median =",Statsmedian(thetaW)	Wavestats /q phiW	Print "Azimuthal angle, phi: mean" ,V_avg,"±",V_sdev,"radians. Median =",Statsmedian(phiW)End//This function will rotate all MTs by two angles (phi and theta). Resulting waves are called rMT*Function Straight(phi,theta)	variable theta,phi		//in radians	String wList=wavelist("MT*",";","")	String wName	Variable i	Make/o zRotationMatrix={{cos(phi),-sin(phi),0},{sin(phi),cos(phi),0},{0,0,1}}	Make/o yRotationMatrix={{cos(theta),0,sin(theta)},{0,1,0},{-sin(theta),0,cos(theta)}}	For (i = 0; i < ItemsInList(wList); i += 1)		wName = StringFromList(i, wList)		String newname="r"+wname		Wave/Z w = $wName		MatrixMultiply w,zRotationMatrix		Wave M_Product		MatrixMultiply M_Product,yRotationMatrix		Duplicate /o M_product $newname	EndForEnd//This function will find the minimum total x-y distance occupied by rMTs after rotation.//It will rotate through a series of angles and find the lowest point.//It then rotates all MTs by these angles using straight()Function LowPoint()		variable theta,phi			//in radians	Make /o /n=90 MatThetaWave	//90° is sufficient with no reflection (and reversal of Mt polarity)	MatThetaWave =x/(180/PI)	Make /o /n=360 MatPhiWave	MatPhiWave =x/(180/PI)		//1° increments seem OK. For 24 MTs, only 0.03 nm difference for increments from lowpoint	Make /o /n=(360,90) MatResWave	String wList=wavelist("MT*",";","")	String wName	Variable i,j,k	Variable px			//pixel value: how much MT (x-y distance) would be seen in gizmo en face	For (j = 0; j < numpnts(MatThetaWave); j +=1)		Theta=MatThetaWave(j)		For (k = 0; k < numpnts(MatPhiWave);  k +=1)			px=0			Phi=MatPhiWave(k)			Make/o zRotationMatrix={{cos(phi),-sin(phi),0},{sin(phi),cos(phi),0},{0,0,1}}			Make/o yRotationMatrix={{cos(theta),0,sin(theta)},{0,1,0},{-sin(theta),0,cos(theta)}}			MatrixMultiply zRotationMatrix,yRotationMatrix			Wave M_Product			Duplicate /O M_Product,zyRotationMatrix				For (i = 0; i < ItemsInList(wList); i += 1)					wName = StringFromList(i, wList)					Wave/Z w = $wName					MatrixMultiply w,zyRotationMatrix					px +=sqrt(((M_Product[1][0]-M_Product[0][0])^2)+((M_Product[1][1]-M_Product[0][1])^2))				EndFor			MatResWave[k][j]=px		EndFor	EndFor	Killwaves zRotationMatrix,yRotationMatrix,zyRotationMatrix,M_Product	///cleanup	WaveStats MatResWave	Print "Rotating z by phi =",V_minRowLoc/(180/PI)," and then rotating y by theta = ",V_minColLoc/(180/PI)	Straight(V_minRowLoc/(180/PI),V_minColLoc/(180/PI))End//This function finds the x,y coordinates of the intersection of mrMT vectors with an xy plane positioned at z=100 nmFunction MakePlane()					//find intersection of mrMTs with plane at z=100	Wave rSCrWave,rSCthetaWave,rSCphiWave	Variable i	Variable n=numpnts(rSCrWave)	Make /O /N=(n,2) rSCxyPlaneWave	//x y values in 2D wave	Variable xb,yb,zb,t				//xa,ya,za are 0,0,0 and t will be 100/zb		for(i = 0;i < n;i +=1)			xb=rSCrWave[i]*(sin(rSCthetaWave[i]))*(cos(rSCphiWave[i]))			yb=rSCrWave[i]*(sin(rSCthetaWave[i]))*(sin(rSCphiWave[i]))			zb=rSCrWave[i]*(cos(rSCthetaWave[i]))			t=100/zb			rSCxyPlaneWave[i][0]=xb*t			rSCxyPlaneWave[i][1]=yb*t	endforEnd
